# Complete Genome Sequence of Stenotrophomonas maltophilia Strain SVIA2, Isolated from Crude Oil-Contaminated Soil in Tabasco, Mexico

**DOI:** 10.1128/MRA.00529-19

**Published:** 2019-07-25

**Authors:** Temidayo Oluyomi Elufisan, Luis Lozano, Patricia Bustos, Isabel Cristina Rodríguez-Luna, Alejandro Sánchez-Varela, Omotayo Opemipo Oyedara, Miguel Ángel Villalobos-López, Xianwu Guo

**Affiliations:** a Laboratorio de Biotecnología Genómica, Centro de Biotecnología Genómica, Instituto Politécnico Nacional, Mexico City, Mexico; b National Center for Technology Management (an agency of the Federal Ministry of Science and Technology), Obafemi Awolowo University, Ife, Osun State, Nigeria; c Centro de Ciencias Genómicas, Universidad Nacional Autónoma de México, Cuernavaca, Morelos, Mexico; d Microbiology Department, Osun State University, Osogbo, Nigeria; e Centro de Investigación en Biotecnología Aplicada, Instituto Politécnico Nacional, Mexico City, Mexico; Loyola University Chicago

## Abstract

Stenotrophomonas maltophilia strain SVIA2 was isolated from crude oil-contaminated soil from Tabasco, Mexico, and displayed a good potential for the degradation of polycyclic aromatic hydrocarbons (PAHs), using naphthalene, anthracene, phenanthridine, or biphenyl as the unique source of carbon. The SVIA2 genome contains essential genes involved in the degradation of PAHs.

## ANNOUNCEMENT

Stenotrophomonas maltophilia has received much attention recently because of its inherent ability to resist a wide range of antimicrobial agents (1). S. maltophilia not only is an opportunistic pathogen but also possesses inherent characteristics to withstand other xenobiotic compounds, such as polycyclic aromatic hydrocarbons (PAHs) (2, 3). S. maltophilia was thus regarded as a versatile bacterium (4). S. maltophilia SVIA2 was isolated from crude oil-contaminated soil retrieved from Tabasco, Mexico. This strain was isolated using *Stenotrophomonas* vancomycin amphotericin B imipenem agar (SVIA) and incubated at 30°C for 48 h. This strain grew effectively in minimal medium (Bushnell Haas medium) with one of the PAHs (naphthalene, anthracene, phenanthridine, or biphenyl) as the unique source of carbon. This implies that the strain could degrade the PAHs for its use.

We sequenced the genome of SVIA2 to understand the genetic basis for its survival in a crude oil-contaminated site and the degradation of PAHs. The genomic DNA was extracted using the Wizard genomic DNA extraction kit (catalog number A1120; Promega Global, USA). The purified genomic DNA was measured using the NanoDrop spectrophotometer and a Qubit 2.0 fluorometer (Thermo Fisher Scientific, Waltham, MA) to verify quality and purity. Whole-genome sequencing was performed using the MiSeq platform (Illumina, Inc., San Diego, CA, USA) according to the standard operation based on a paired-end library and a mate pair library of 5-kb fragments. The genomic DNA library was prepared using the Nextera Flex DNA library preparation kit. The resulting sequence generates a total of 10,957,743 raw reads amounting to about 300× genome coverage. The reads were inspected for data quality using FastQC v0.11.3 (Babraham Institute, Cambridge, United Kingdom; https://www.bioinformatics.babraham.ac.uk/projects/fastqc/) using the default parameters and then trimmed with Trim Galore v0.4.4 (https://www.bioinformatics.babraham.ac.uk/projects/trim_galore/) to remove low-quality reads and adaptors using the command “trim_galore –fastqc –paired –retain unpaired read1.fastq read2.fastq -o SVIA2.” The reads were then assembled *de novo* using SPAdes v3.1.12 with the command parameter “spades.py -k 50, 70, 90, 127 –careful” (5). The *de novo* assembly resulted in 69 contigs. The resulting contigs were reduced into one scaffold and one contig with MeDuSa (6) using S. maltophilia K279a as the reference genome. The resulting genome sequence of S. maltophilia SVIA2 is 4,501,027 bp in length. The overall G+C content of the assembled genome is 66.63%.

The Prokka genome annotation pipeline v1.12 was used for annotation of the SVIA2 genome (7). It contains 4,028 putative coding sequences (CDS), 5 rRNA genes, and 77 tRNAs. Many genes that could be involved in the degradation of PAHs, such as the genes encoding *todB*-dependent protein 2-octaprenyl 6-methoxyphenol hydroxylase, haloalkane dehalogenase, and 4-hydroxyphenylpyruvate dioxygenase, were present in SVIA2. Others include genes encoding *S*-(hydroxymethyl) glutathione dehydrogenase I, involved in the metabolism of methane, and salicylate hydroxylase (*nahG*), which hydroxylates salicylic aldehyde to form catechol (8). Also present in the genome are genes essential for the cleavage of aromatic rings, such as alcohol dehydrogenase, isoquinoline I oxidoreductase, and the lyase-encoding genes, which are necessary for the conversion of salicylate to catechol (9). The genome has 29 genomic island (GI) regions, as predicted by IslandViewer 4 (10), which contained some genes involved in the degradation of PAH.

The genome of S. maltophilia SVIA2 is rich in genes essential for the degradation of PAHs and therefore could be an excellent tool for bioremediation. This genome will provide useful information to study the mechanism employed by Stenotrophomonas spp. in the degradation of PAHs.

### Data availability.

The nucleotide sequence is available at DDBJ/EMBL/GenBank under the accession number CP033586. The raw sequence data are available at SRP198817.
